# Renal Single-Cell RNA Sequencing and Digital Cytometry in Dogs with X-Linked Hereditary Nephropathy

**DOI:** 10.3390/ani15142061

**Published:** 2025-07-12

**Authors:** Candice P. Chu, Daniel Osorio, Mary B. Nabity

**Affiliations:** 1 Department of Veterinary Pathobiology, College of Veterinary Medicine & Biomedical Sciences, Texas A&M University, College Station, TX 77843, USA; 2 Department of Veterinary Integrative Biosciences, College of Veterinary Medicine & Biomedical Sciences, Texas A&M University, College Station, TX 77843, USA

**Keywords:** Alport syndrome, digital cytometry, dog, kidney, RNA-seq, scRNA-seq, X-linked hereditary nephropathy

## Abstract

Chronic kidney disease (CKD) significantly affects canine health, but the precise cellular mechanisms of this condition remain elusive. In this study, we used single-cell RNA sequencing (scRNA-seq) to profile renal cellular gene expression in a canine model of X-linked hereditary nephropathy (XLHN). Dogs with this condition exhibit juvenile-onset CKD similar to that seen in human Alport syndrome. Post-mortem renal cortical tissues from an affected male dog and a heterozygous female dog were processed to obtain single-cell suspensions. In total, we recovered up to 13,190 cells and identified 11 cell types, including major kidney cells and immune cells. Differential gene expression analysis comparing the affected male and heterozygous female dogs identified cell-type specific pathways that differed in a subpopulation of proximal tubule cells. These pathways included the integrin signaling pathway and the pathway for inflammation mediated by chemokine and cytokine signaling. Additionally, using machine learning-empowered digital cytometry, we deconvolved bulk mRNA-seq data from a previous canine study, revealing changes in cell type proportions across CKD stages. These results underline the utility of single-cell methodologies and digital cytometry in veterinary nephrology.

## Simple Summary

Chronic kidney disease (CKD) is common in dogs, yet understanding the specific cellular changes in this condition remains challenging. In this study, we used single-cell RNA sequencing (scRNA-seq) to analyze kidney tissues from dogs with X-linked hereditary nephropathy (XLHN), a naturally occurring model of canine CKD. We identified differentially expressed genes in the pathways linked to podocyte dysfunction and tubular inflammation. These findings were validated using digital cytometry in additional dogs. The integration of these advanced techniques enhances our understanding of CKD progression in dogs and helps in identifying potential therapeutic targets.

## 1. Introduction

Chronic kidney disease (CKD) is a significant cause of morbidity and mortality in all breeds of dogs [1]. While many cases of canine CKD are caused by underlying glomerular diseases, some breeds of dogs have hereditary defects that precipitate renal failure at a young age [2,3]. For example, dogs with X-linked hereditary nephropathy (XLHN) have a 10-base-pair deletion in the α5 chain of the type IV collagen (COL4A5) gene on the X chromosome. This deletion leads to an alteration in the structure and function of the glomerular basement membrane. This results in CKD that rapidly progresses to end-stage renal disease by one year of age in affected (hemizygous) males and by variable ages in heterozygous (carrier) females [3]. This naturally occurring canine model has been studied as an example of canine CKD caused by glomerular disease and as an animal model of human Alport syndrome.

Previous studies have used PCR [4,5,6], microarrays [5], and RNA-sequencing (RNA-seq) [7,8] to characterize the differential gene and microRNA expression in the renal tissues of XLHN dogs. However, in traditional bulk tissue mRNA-seq, global gene expression may be at risk of being masked by outlier cells and the overrepresentation of specific cells [9]. The recent development of single-cell RNA-seq (scRNA-seq) technologies and digital cytometry can overcome this limitation.

By elucidating gene expression in individual cells, scRNA-seq provides unprecedented resolution, allowing the classification of cell types and the construction of comprehensive atlases that reflect the innate heterogeneity of the kidney [10]. scRNA-seq has been used to profile cells in mouse kidneys, helping to identify cellular targets involved in kidney disease [11]. Additionally, scRNA-seq has allowed the identification of distinct receptors and signal ligands in different cells, enhancing our understanding of renal pathophysiology [12]. For example, in mice with CKD, scRNA-seq has identified in renal tubules the decreased expression in the TGF-beta pathway of an adaptor protein that would protect mice from CKD [13]. In humans with systemic lupus erythematosus, Type I interferon-inducible genes in renal tubular cells have been correlated with chronicity index, IgG deposition, and the degree of proteinuria [14].

Moreover, the deconvolution of mRNA-seq using scRNA-seq data can further characterize the cell composition of previously studied tissue samples and can precisely identify the cell types with altered gene expression that might contribute uniquely to disease progression. CIBERSORTx, a web-based machine learning algorithm, can perform “digital cytometry” to deconvolve bulk mRNA-seq data, achieving *in silico* tissue dissection to indicate cell composition [15]. In studies of mouse and human CKD, digital cytometry has been used to estimate the proportion of immune and non-immune cells based on the bulk RNA-sequencing data of kidneys [16,17,18]. However, to the best of our knowledge, scRNA-seq research has not been previously used in a large animal model of Alport syndrome.

The goal of this pilot study was to profile the RNA expression of individual kidney cells in a canine model of CKD using scRNA-seq technologies and to leverage the generated scRNA-seq data to deconvolve the bulk mRNA-seq data, achieving digital cytometry. This study provides new insights into the current understanding of pathogenesis and disease progression of Alport syndrome. It also facilitates the identification and characterization of previously unrecognized cell states, differentially expressed genes, and molecular pathways involved in disease mechanisms, thus potentially contributing to both canine and human health.

## 2. Materials and Methods

### 2.1. Sample Collection

The dogs in this study were part of a colony of XLHN dogs maintained at Texas A&M University [19]. The dogs, which were untreated, were in unrelated studies approved by the Texas A&M University Institutional Animal Care and Use Committee (protocol IACUC 2018-0195). One gram of fresh kidney cortex was collected immediately after the euthanasia of an affected male dog and a heterozygous female dog with XLHN that had reached their clinical endpoint (serum creatinine concentration ≥ 5 mg/dL).

### 2.2. Single-Cell Suspension Preparation

The time from kidney tissue collection to single-cell suspension was less than 30 minutes. For tissue dissociation, the sample was placed in 20 mL of RPMI solution (Thermo Fisher Scientific, Waltham, MA, USA) and then transferred into a gentleMACS C tube (Miltenyi Biotec, Bergisch Gladbach, Germany) containing an enzyme mix from the MACS Multi Tissue Dissociation Kit 1 (Miltenyi Biotec, Bergisch Gladbach, Germany). The sample was dissociated inside the gentleMACS C tube through a gentleMACS Octo Dissociator (Miltenyi Biotec, Bergisch Gladbach, Germany) using the built-in program 37C_Multi_B. To ensure that the single-cell suspension had no debris, had an optimal concentration, and contained viable cells, the 10x Genomic guidelines for optimal sample preparation were followed [20]. First, cells were filtered through a MACS SmartStrainer (70 μm) (Miltenyi Biotec, Bergisch Gladbach, Germany) and centrifuged at 300× *g* for 7 min to collect cell pellets and ensure that the single-cell suspension was free of debris. The nucleated cell count was determined in duplicate using a Countess Cell Counter (Thermo Fisher Scientific, Waltham, MA, USA), to verify that the cell concentration was 700–1200 cells/μL, as recommended by the sequencing facility. Finally, dead cells were removed using a MACS Dead Cell Removal Kit (Miltenyi Biotec, Bergisch Gladbach, Germany) in conjunction with an MS column (Miltenyi Biotec, Bergisch Gladbach, Germany) that contains magnetic beads for cell binding.

### 2.3. Single-Cell RNA Sequencing

We used 10x Genomics as the scRNA-seq platform because of its high throughput, sequencing depth, high sensitivity, and compatibility with the Illumina system [9]. Preparation of the single-cell suspension was immediately followed by library preparation using a 10x Genomics Chromium Single Cell 3′ Reagent Kit (v3 Chemistry) (10x Genomics, Pleasanton, CA, USA). The cDNA was sequenced with Illumina NovaSeq 6000 (Illumina, San Diego, CA, USA) (2 × 150) to reach a target sequencing depth (raw reads count) of 20,000 reads per cell with a maximum input of 10,000 cells.

### 2.4. Sample Size Calculation

Prospective sample size calculation was performed using the Single-Cell One-sided Probability Interactive Tool (SCOPIT, v1.1.4) [21]. The parameters were based on the renal scRNA-seq data of one affected male dog: number of cells of each subpopulation which must be sequenced = 92, required probability of sequencing this many cells from each subpopulation = 0.8, and number of subpopulations with the lowest frequency = 0.05. Therefore, at least 9615 cells were required for a 0.8 probability of success in sequencing at least 92 cells from each identified subpopulation.

### 2.5. Data and Statistical Analysis

After the acquisition of raw data, bustool (0.40.0) was used for pre-processing, and Kallisto (0.46.2) was used for pseudoalignment with canine genome assembly ROS_Cfam_1.0 [22]. DropletUtils (1.10.3) was used to filter empty droplets following best-practice guidelines [23,24,25,26]. Seurat (4.1.1) was used for single-cell data analysis [27]. Dimension reduction was performed using uniform manifold approximation and projection (UMAP). Cell marker genes were defined as present if they appeared in at least 25% of the cells within the cluster with a log fold change threshold of 0.25. Differential expression was assessed using the Wilcoxon Rank Sum test (adjusted *p* value < 0.05). Manual cell annotation was performed using the single-cell databases, CellMarker 2.0 [28] and PanglaoDB [29], and published human and mouse kidney data [30,31,32].

Protein Analysis Through Evolutionary Relationships (PANTHER) pathway analysis was performed using the canine genome in the PANTHER Overrepresentation Test (released 26 February 2024) in PANTHER version 18.0 (http://www.pantherdb.org/, released 1 August 2023). Fisher’s exact test with Bonferroni’s correction for multiple testing was used. Statistical significance was set at a Bonferroni-corrected *p* value < 0.05.

For the deconvolution of bulk RNA-seq data, the scRNA-seq data in the affected male dog was uploaded into CIBERSORTx, a digital cytometry algorithm, with default settings [33]. This algorithm inferred cell type abundance in bulk mRNA-seq data obtained from 6 XLHN dogs and 2 age-matched control dogs [7], thereby indicating cell composition without physical dissociation and enabling cost-effective high-throughput tissue profiling. ANOVA was used to examine the differences in cell population between affected and control dogs at three clinical time points. Statistical significance was set at *p* value < 0.001.

## 3. Results

### 3.1. Histopathological Evaluation of Kidney Biopsies

**Figure 1:**
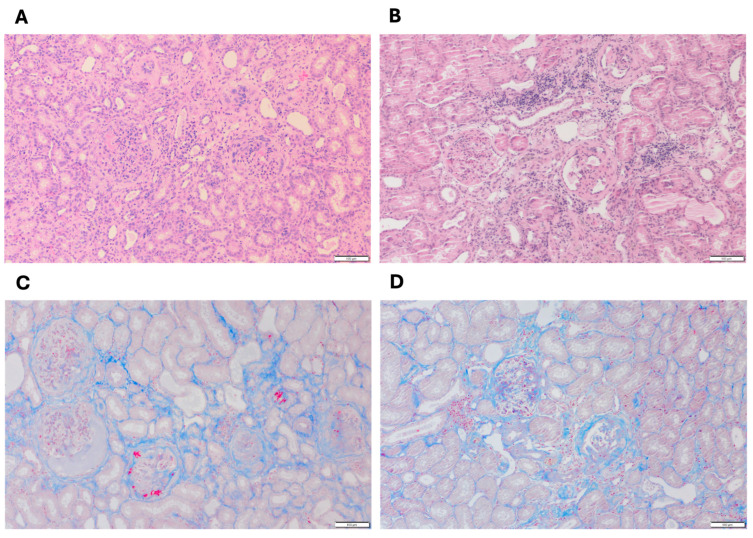
Representative renal cortical histopathology from (**A**,**C**) an affected male dog and (**B**,**D**) a heterozygous female dog with end-stage XLHN. (**A**,**B**) Hematoxylin and eosin (H&E) stain; (**C**,**D**) Masson’s trichrome stain; magnification, 10×; scale bar: 100 µm.

Figure 1 shows the representative histological features of cortical kidney tissues from dogs with end-stage XLHN (serum creatinine levels ≥ 5 mg/dL). In the affected male dog (Figure 1A,C), extensive tubular atrophy, interstitial fibrosis, and a pronounced infiltration of inflammatory cells were observed, accompanied by prominent glomerulosclerosis [7]. In contrast, the heterozygous female dog (Figure 1B,D) exhibited comparatively milder changes, including moderate tubular degeneration, less severe interstitial fibrosis, and fewer inflammatory infiltrates, consistent with a less severe disease phenotype.

### 3.2. Unbiased Classification of Canine Renal Cells Using scRNA-Seq

**Figure 2:**
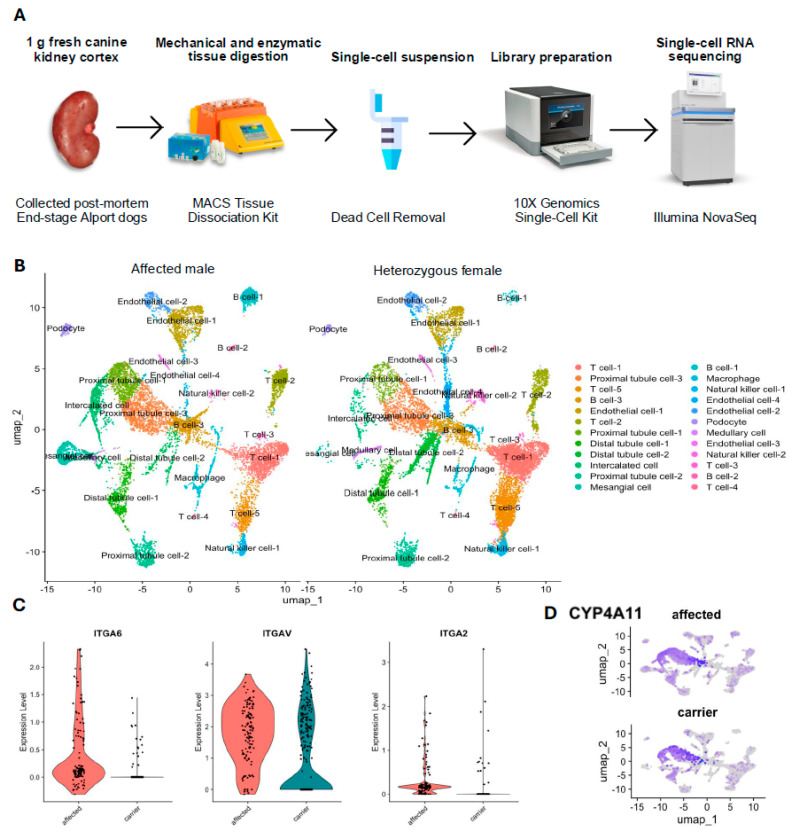
Single-cell transcriptome atlas of renal cells. (**A**) Single-cell RNA-sequencing workflow. (**B**) Uniform manifold approximation and projection (UMAP) visualization of 24 cell types in the integrated dataset of affected male dog and heterozygous female dog with X-linked hereditary nephropathy (XLHN). This plot displays a comprehensive comparison of all renal cells using UMAP analysis to illustrate the distribution of various cell types and their subpopulations in both the affected male (**left**) and the heterozygous female (**right**). Each color represents a different cell type, and annotations indicate their spatial distribution and density. (**C**) Comparative expression levels of integrin genes ITGA6, ITGAV, and ITGA2 in the podocytes of the affected male (red) and heterozygous (carrier) female (teal). The violin plots illustrate the distribution and median expression levels of each gene. Each dot represents an individual podocyte within the group. (**D**) Enrichment of cytochrome P450 family 4 subfamily A member 11 (CYP4A11) in a subgroup of proximal tubule cells (proximal tubule cell-2) on the UMAP plots of the affected male (affected) and heterozygous female (carrier).

The kidney cortex tissues of the affected male dog and heterozygous female dog were subjected to scRNA-seq analysis to obtain cellular gene expression data (Figure 2A). A summary of the sequencing data from these two dogs appears in Table 1. Using the principal component analysis in Seurat, we identified 24 cell clusters in the integrated dataset and visualized them in UMAP. After manual annotation using marker genes listed in Table 2, 11 unique cell types (with subtypes) were identified in both samples (Figure 2B). Subpopulations of each cell type were also identified, and these subpopulations were kept separate for further downstream analysis. Table 2 shows the proportion of each cell type.

### 3.3. Differential Gene Expression and Pathway Analysis

The overall comparison between the affected male and the heterozygous female revealed 217 differentially expressed genes within the tissue as a whole. In the PANTHER pathway analysis, the FAS signaling pathway, CCKR signaling map, and integrin signaling pathway were enriched 12.84-, 4.66-, and 4.23-fold, respectively, within all differentially expressed genes (Table 3). On the cellular level, when comparing podocyte-specific gene expression between the affected male and the heterozygous female, 482 genes were differentially expressed, including 21 genes that were enriched 4.52-fold in the integrin signaling pathway (Table 3). Figure 2C shows the expression of three signature genes in the integrin signaling pathway (ITGA6, ITGAV, and ITGA2) in podocytes from each dog. In a subpopulation of proximal tubule cells, the proximal tubule cells-2, there were 55 genes expressed differentially by the affected male and the heterozygous female, including five genes in the inflammation pathway mediated by chemokine and cytokine signaling, for which there was an 8.45-fold enrichment (Table 3). Compared with the other two proximal tubule cell subpopulations, proximal tubule cells-2 showed a significant 9.34-fold upregulation in cytochrome P450 family 4 subfamily A member 11 (CYP4A11) (*p* = 2.14 × 10 ^−190^) (Figure 2D).

### 3.4. Digital Cytometry Using Bulk mRNA-Seq Data

**Figure 3:**
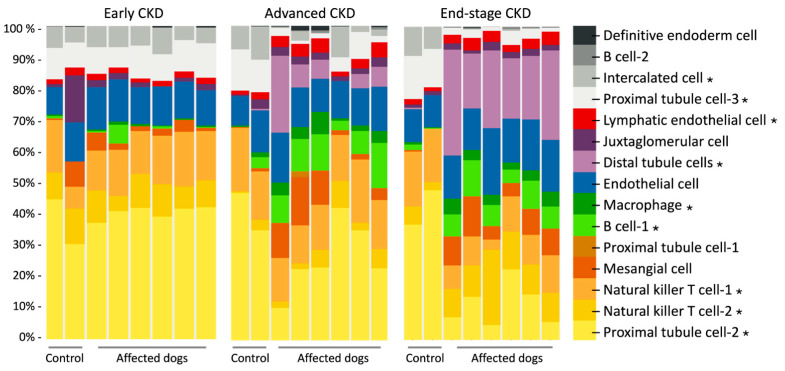
Deconvolved bulk mRNA-seq data from six male dogs with X-linked hereditary nephropathy (XLHN) and two age-matched same-sex littermates. The bar charts show how cell type proportion changed during disease progression. The asterisks denote cell types that differed in proportion between affected and control dogs at three clinical time points (*p* < 0.001).

Using renal bulk RNA-seq data on previously analyzed kidney cortical tissue from XLHN dogs and our current scRNA-seq data, we used CIBERSORTx to estimate the abundance of renal cell types. The deconvolved bulk mRNA-seq data showed that the cell type proportions changed as canine XLHN progressed (Figure 3). The cell types that displayed significant changes under the ANOVA test (*p* < 0.001) were B cell-1, distal tubule cell, intercalated cell, lymphatic endothelial cell, macrophage, natural killer T cell-1, natural killer T cell-2, proximal tubule cell-2, and proximal tubule cell-3.

## 4. Discussion

XLHN dogs have been studied as a naturally occurring model of canine CKD and a large animal model of human Alport syndrome. The differential gene expression, molecular pathways, and microRNA changes involved in the progression of this disease have been studied on a bulk-tissue basis [7,8]. This study aimed to apply scRNA-seq to characterize the cell populations present in the renal cortex of XLHN dogs. Through the comparison of scRNA-seq data in an affected male and a heterozygous female, the identification of differentially expressed genes and the corresponding overrepresented pathways in specific cell types can provide unprecedented resolution in understanding the pathogenesis of canine CKD.

In this study, renal cortical tissue from an affected male and a heterozygous female dog were processed, recovering up to 13,190 high-quality cells, which exceeded the required cell number from our sample size calculation. The clustering analysis identified the same number of cell clusters within each sample, with T cells being the most prominent cell type (Figure 2B). In human patients and a mouse model of Alport syndrome, T cell influx is crucial to disease progression [34]. Our finding of T cell enrichment is consistent with that from the previous bulk RNA-seq gene expression analysis and immunostaining of kidney tissue from XLHN dogs with end-stage renal disease [7]. Moreover, the histopathology from a larger cohort of XLHN dogs (n = 9) demonstrated diffuse lymphocytic infiltration of the renal interstitium [19]. Although the lymphocyte population was not phenotyped, this could support that lymphocyte predominance is reproducible across multiple affected individuals and not limited to the present single-cell dataset.

Podocytes are the primary source of α3/4/5 type IV collagen in the glomerular basement membrane [35]. When comparing podocyte-specific gene expression between the affected male and heterozygous female dogs in our study, differential expressions were observed in three key integrin signaling genes: ITGA6, ITGAV, and ITGA2 (Figure 2C). The expression of ITGA6 was elevated in the affected male, while ITGAV was expressed at comparable levels in both dogs, albeit slightly higher in the affected male. Integrin signaling is known to contribute to podocyte injury and disease progression in CKD. In mouse models of diabetic nephropathy, crosstalk between the TGF-β, Wnt/β-catenin, and integrin signaling pathways contributed to podocyte injury and dysfunction, exacerbating the progression of CKD [36,37,38,39]. Furthermore, the blockade of αVβ3 integrin, a receptor encoded by the ITGAV gene, has been reported to attenuate albuminuria and renal histologic damage in both diabetic rat and pig models, highlighting the functional importance of integrin-mediated cell adhesion and signaling in CKD [40,41]. In our data, ITGA2 expression was markedly reduced in the heterozygous female relative to the affected male, with only minimal expression observed. ITGA2 encodes a subunit of the α2β1 integrin, a major collagen-binding receptor. The deletion of ITGA2 in Col4a3-deficient Alport mice resulted in significantly improved renal outcomes, including reduced proteinuria, lower blood urea nitrogen concentrations, decreased extracellular matrix deposition, and a 20% increase in survival [42]. These findings underscore a pathogenic role of collagen-binding integrins in Alport syndrome. Reduced ITGA2 expression may contribute to slower disease progression in heterozygous females, and the inhibition of collagen receptors may offer a novel strategy to treat patients with Alport syndrome.

Capturing transcriptional changes within cell subpopulations can be critical for identifying cell-specific responses to drugs and uncovering crucial regulators, thereby enabling therapies targeted to these subpopulations [43,44]. In our study, the data obtained from all cell subpopulations was used to determine specific cell markers for cell identification. In the proximal tubule cell-2 population, the inflammation mediated by the chemokine and cytokine signaling pathway was greater in the affected male than in the heterozygous female. Included in this pathway is CCL2, a pro-inflammatory cytokine that participates in recruiting monocytes and T cells to the site of tissue injury [45]. The expression of CCL2 is typically observed in immune cells, such as macrophages, in membranous nephropathy [46]. However, during a late stage of kidney disease, injured proximal tubule cells can enter a profibrotic, pro-inflammatory status via the expression of CCL2 and the activation of the NF-κB, TNF-, and AP-1 signaling pathways, thus likely serving as contributors to CKD progression [47].

In this study, previously obtained bulk mRNA-seq data was refined with scRNA-seq to characterize how cell composition changed through the progression of CKD using digital cytometry. We identified several cell clusters with statistically significant changes in abundance over time (Figure 3). Of particular note was proximal tubule cell-2, for which the expression in the control group remained almost constant over time, but a substantial decrease toward end-stage CKD in the affected dogs with XLHN was also shown. In the proximal tubule cell-2 cluster, CYP4A11 was significantly upregulated relative to that in the proximal tubule cell-1 and cell-3 clusters (Figure 2D). An integrated analysis of five CKD transcriptome datasets has identified CYP4A11 as being underexpressed across nine CKD entities [48]. CYP4A11 is an enzyme responsible for oxidizing erythrodonic acid into 20-hydroxyeicosatetraenoic acid (20HETE), which has an antihypertensive effect [49]. The observed decrease in the proximal tubule cell-2 cluster, along with its enrichment in CYP4A11, suggests a reduction in protective effects that may contribute to CKD progression in XLHN dogs.

We recognize the limitations inherent to our study design. First, the sample size of only one affected male dog and one heterozygous female dog and the confounding by sex differences are major limitations. Sex differences can affect baseline gene expression and cell type proportions, even under normal conditions [50]. Thus, the observed transcriptional differences may partially reflect inherent sex-related expression rather than genotype or disease state alone. Second, the scRNA-seq data were generated in two separate sequencing runs, introducing potential batch effects from technical discrepancies between runs (e.g., differences in sequencing depth and library preparation). Although we minimized batch-related bias using Seurat’s data integration pipeline [27]—an anchor-based method designed to correct technical differences while preserving biological signals—residual batch effects may still influence the results. Third, the analysis was performed only at end-stage disease, which might preclude insights into interventions relevant to early disease.

To the best of the authors’ knowledge, this pilot study is the first attempt to profile cellular gene expression in a large animal model of Alport syndrome. In this study, renal scRNA-seq data from XLHN dogs were used to quantify cell type proportions, which were then used to infer the cell type abundance of a previously published study using digital cytometry. The findings can facilitate the discovery of genes and cell types relevant to disease progression in canine CKD and help to identify potential therapeutic targets for Alport syndrome.

## 5. Conclusions

In conclusion, this pilot study supports the value of integrating scRNA-seq and digital cytometry in elucidating the cellular mechanisms underlying CKD progression in XLHN dogs. Our findings revealed distinct gene expression patterns and identified cell-type-specific molecular pathways, particularly those associated with podocyte dysfunction and tubular inflammation, offering novel insights into CKD pathogenesis. Although limited by sample size, these results establish a foundation for further research and highlight potential therapeutic targets with translational relevance to both veterinary and human nephrology. Future studies incorporating larger cohorts are warranted to evaluate these findings and enhance their clinical applicability.

## Figures and Tables

**Table 1:** Summary of scRNA-seq data.

	Affected Male Dog	Heterozygous Female Dog
Reads mapped to genome	95.4%	95.3%
Number of cells examined	12,778	13,190
Median genes per cell	1575	1278
Total genes detected	25,438	24,208

**Table 2:** Marker genes, numbers, and percentages of each cell subpopulation.

Cell Type	Marker Genes	Cell Number in Affected Male Dog	Cell Percentage in Affected Male Dog	Cell Number in Heterozygous Female Dog	Cell Percentage in Heterozygous Female Dog
B cell-1	CD79B [31]	364	2.85%	394	2.99%
B cell-2	CD79B [31] CTSS [32]	9	0.07%	142	1.08%
B cell-3	(Determined by CellMarker [28])	908	7.11%	853	6.47%
Distal tubule cell-1	UMOD, DEFB1, TMEM213 [31]	650	5.09%	573	4.34%
Distal tubule cell-2	DEFB1, ATP6V1G3 [31]	205	1.60%	918	6.96%
Endothelial cell-1	PECAM1, EMCN, FLT1, TEK [30] FAM167B, SOX17, MYCT1, TIE1, ADGRL4, SOX18, PTPRB [32]	910	7.12%	717	5.44%
Endothelial cell-2	PECAM1, EMCN, FLT1, TEK, ADGRL4, SOX18 [30] FAM167B, SOX17, MYCT1, TIE1, PTPRB [32]	238	1.86%	313	2.37%
Endothelial cell-3	PECAM1, EMCN [30] GPHBP1, MYCT1, TIE1, SOX18 [32]	160	1.25%	150	1.14%
Endothelial cell-4	PECAM1, EMCN [30] GPHBP1, MYCT1, TIE1, SOX18 [32]	103	0.81%	524	3.97%
Intercalated cell	ATP6V1G3, ATP6V0D2, TMEM213 [31]	843	6.60%	223	1.69%
Macrophage	LYZ, CD14 [31] C1QB, CTSS, CCL4, CCL3, AIF1, CX3CR1 [32]	182	1.42%	495	3.75%
Medullary cell	FBLN5 [28]	71	0.56%	268	2.03%
Mesangial cell	MYLL9, ITGA8 [30]	909	7.11%	37	0.28%
Natural killer cell-1	NKG7 [31] CD52, CCL4 [32]	857	6.71%	50	0.38%
Natural killer cell-2	NKG7 [31] CD52 [32]	40	0.31%	212	1.61%
Podocyte	NPHS2, NPHS1, WT1, SYNPO [30]	281	2.20%	66	0.50%
Proximal tubule cell-1	SLC13A3, SLC34A1, GPX3 [31] RDH16 [32]	1,031	8.07%	347	2.63%
Proximal tubule cell-2	SLC13A3, SLC34A1, GPX3 [31] RDH16 [32]	458	3.58%	523	3.97%
Proximal tubule cell-3	GPX3 [31] RDH16 [32]	1,431	11.20%	1182	8.96%
T cell-1	IL7R [31] CD52 [32]	1,586	12.41%	2863	21.71%
T cell-2	IL7R [31] CD52 [32]	781	6.11%	620	4.70%
T cell-3	IL7R [31] NKG7 [31] CCL4 [32]	95	0.74%	66	0.50%
T cell-4	IL7R [31] CTSS, CX3CR1 [32]	112	0.88%	20	0.15%
T cell-5	CCL4, CCL3 [32]	554	4.34%	1634	12.39%

A reference for each marker gene is provided.

**Table 3:** Overrepresented PANTHER pathways in differentially expressed genes in a male dog with X-linked hereditary nephropathy (XLHN) and a heterozygous female dog.

Cell Types	PANTHER Pathways	Genes in the Pathway	Fold Enrichment	*p* Value
All cells	FAS signaling pathway	LMNA, ENSCAFG00845026419, JUN, GSN	12.84	3.82 × 10^−2^
	CCKR signaling map	CPE, CREM, CCK, JUN, MMP9, IER3, CCK, MEF2C	4.66	2.24 × 10^−2^
	Integrin signaling pathway	COL1A1, COL1A2, ACAT2, COL6A3, COL1A2, FN1, COL3A1, COL1A1, FN1	4.23	4.62 × 10^−2^
Podocytes	Integrin signaling pathway	COL11A1, COL4A1, ITGAV, VCL, COL1A2, ACTA2, ASAP1, COL6A3, NTN4, CAV1, COL1A2, COL5A2, FN1, COL8A2, ITGA2, LAMB1, COL4A2, FN1, COL4A4, ITGA6, COL5A3	4.52	1.47 × 10^−6^
Proximal tubule cell-2	Inflammation mediated by chemokine and cytokine signaling pathway	CCL2, MYH11, ACTA2, ITGA2, CXCL10	8.45	1.14 × 10^−2^

## Data Availability

The raw data supporting the conclusions of this article will be made available by the authors on request.

## Abbreviations

The following abbreviations are used in this manuscript:

20HETE	20-hydroxyeicosatetraenoic acid
CKD	Chronic kidney disease
COL4A5	α5 chain of the type IV collagen
CYP4A11	Cytochrome P450 family 4 subfamily A member 11
PANTHER	Protein Analysis THrough Evolutionary Relationships
RNA-seq	RNA sequencing
scRNA-seq	Single-cell RNA sequencing
XLHN	X-linked Hereditary Nephropathy

## References

[1] O’Neill D.G., Elliott J., Church D.B., McGreevy P.D., Thomson P.C., Brodbelt D.C. (2013). Chronic Kidney Disease in Dogs in UK Veterinary Practices: Prevalence, Risk Factors, and Survival. J. Vet. Intern. Med..

[2] Zheng K., Thorner P.S., Marrano P., Baumal R., McInnes R.R. (1994). Canine X Chromosome-Linked Hereditary Nephritis: A Genetic Model for Human X-Linked Hereditary Nephritis Resulting from a Single Base Mutation in the Gene Encoding the Alpha 5 Chain of Collagen Type IV. Proc. Natl. Acad. Sci. USA.

[3] Cox M.L., Lees G.E., Kashtan C.E., Murphy K.E. (2003). Genetic Cause of X-Linked Alport Syndrome in a Family of Domestic Dogs. Mamm. Genome.

[4] Benali S.L., Lees G.E., Nabity M.B., Arico A., Drigo M., Gallo E., Giantin M., Aresu L. (2016). X-Linked Hereditary Nephropathy in Navasota Dogs: Clinical Pathology, Morphology, and Gene Expression During Disease Progression. Vet. Pathol..

[5] Greer K.A., Higgins M.A., Cox M.L., Ryan T.P., Berridge B.R., Kashtan C.E., Lees G.E., Murphy K.E. (2006). Gene Expression Analysis in a Canine Model of X-Linked Alport Syndrome. Mamm. Genome.

[6] Rao V.H., Lees G.E., Kashtan C.E., Nemori R., Singh R.K., Meehan D.T., Rodgers K., Berridge B.R., Bhattacharya G., Cosgrove D. (2003). Increased Expression of MMP-2, MMP-9 (Type IV Collagenases/Gelatinases), and MT1-MMP in Canine X-Linked Alport Syndrome (XLAS). Kidney Int..

[7] Chu C.P., Hokamp J.A., Cianciolo R.E., Dabney A.R., Brinkmeyer-Langford C., Lees G.E., Nabity M.B. (2017). RNA-Seq of Serial Kidney Biopsies Obtained during Progression of Chronic Kidney Disease from Dogs with X-Linked Hereditary Nephropathy. Sci. Rep..

[8] Chu C.P., Liu S., Song W., Xu E.Y., Nabity M.B. (2021). Small RNA Sequencing Evaluation of Renal microRNA Biomarkers in Dogs with X-Linked Hereditary Nephropathy. Sci. Rep..

[9] Oliverio A.L., Bellomo T., Mariani L.H. (2019). Evolving Clinical Applications of Tissue Transcriptomics in Kidney Disease: A Mini-Review. Front. Pediatr..

[10] Saliba A.E., Westermann A.J., Gorski S.A., Vogel J. (2014). Single-Cell RNA-Seq: Advances and Future Challenges. Nucleic Acids Res..

[11] Park J., Shrestha R., Qiu C., Kondo A., Huang S., Werth M., Li M., Barasch J., Susztak K. (2018). Single-Cell Transcriptomics of the Mouse Kidney Reveals Potential Cellular Targets of Kidney Disease. Science.

[12] Chen L., Lee J.W., Chou C.L., Nair A.V., Battistone M.A., Paunescu T.G., Merkulova M., Breton S., Verlander J.W., Wall S.M. (2017). Transcriptomes of Major Renal Collecting Duct Cell Types in Mouse Identified by Single-Cell RNA-Seq. Proc. Natl. Acad. Sci. USA.

[13] Qiu C., Huang S., Park J., Park Y., Ko Y.A., Seasock M.J., Bryer J.S., Xu X.X., Song W.C., Palmer M. (2018). Renal Compartment-Specific Genetic Variation Analyses Identify New Pathways in Chronic Kidney Disease. Nat. Med..

[14] Der E., Ranabothu S., Suryawanshi H., Akat K.M., Clancy R., Morozov P., Kustagi M., Czuppa M., Izmirly P., Belmont H.M. (2017). Single Cell RNA Sequencing to Dissect the Molecular Heterogeneity in Lupus Nephritis. JCI Insight.

[15] Newman A.M., Liu C.L., Green M.R., Gentles A.J., Feng W., Xu Y., Hoang C.D., Diehn M., Alizadeh A.A. (2015). Robust Enumeration of Cell Subsets from Tissue Expression Profiles. Nat. Methods.

[16] Chew C., Brand O.J., Yamamura T., Lawless C., Morais M.R.P.T., Zeef L., Lin I.-H., Howell G., Lui S., Lausecker F. (2024). Kidney Resident Macrophages Have Distinct Subsets and Multifunctional Roles. Matrix Biol..

[17] Bai F., Wang C., Fan X., Fang L., Li L., Zhang X., Yu K., Liu L., Guo L., Yang X. (2024). Novel Biomarkers Related to Oxidative Stress and Immunity in Chronic Kidney Disease. Heliyon.

[18] Dhillon P., Mulholland K.A., Hu H., Park J., Sheng X., Abedini A., Liu H., Vassalotti A., Wu J., Susztak K. (2023). Increased Levels of Endogenous Retroviruses Trigger Fibroinflammation and Play a Role in Kidney Disease Development. Nat. Commun..

[19] Lees G.E., Helman R.G., Kashtan C.E., Michael A.F., Homco L.D., Millichamp N.J., Camacho Z.T., Templeton J.W., Ninomiya Y., Sado Y. (1999). New Form of X-Linked Dominant Hereditary Nephritis in Dogs. Am. J. Vet. Res..

[20] 10x Genomics Chromium Single Cell Applications—Guidelines for Optimal Sample Preparation. https://www.10xgenomics.com/support/single-cell-gene-expression/documentation/steps/sample-prep/chromium-single-cell-applications-guidelines-for-optimal-sample-preparation.

[21] Davis A., Gao R., Navin N.E. (2019). SCOPIT: Sample Size Calculations for Single-Cell Sequencing Experiments. BMC Bioinform..

[22] Du Y., Huang Q., Arisdakessian C., Garmire L.X. (2020). Evaluation of STAR and Kallisto on Single Cell RNA-Seq Data Alignment. G3 (Bethesda).

[23] Lun A.T.L., Riesenfeld S., Andrews T., Dao T.P., Gomes T., Marioni J.C., Participants in the 1st Human Cell Atlas Jamboree (2019). EmptyDrops: Distinguishing Cells from Empty Droplets in Droplet-Based Single-Cell RNA Sequencing Data. Genome Biol..

[24] Griffiths J.A., Richard A.C., Bach K., Lun A.T.L., Marioni J.C. (2018). Detection and Removal of Barcode Swapping in Single-Cell RNA-Seq Data. Nat. Commun..

[25] Zheng G.X.Y., Terry J.M., Belgrader P., Ryvkin P., Bent Z.W., Wilson R., Ziraldo S.B., Wheeler T.D., McDermott G.P., Zhu J. (2017). Massively Parallel Digital Transcriptional Profiling of Single Cells. Nat. Commun..

[26] Luecken M.D., Theis F.J. (2019). Current Best Practices in Single-Cell RNA-Seq Analysis: A Tutorial. Mol. Syst. Biol..

[27] Hao Y., Hao S., Andersen-Nissen E., Mauck W.M., Zheng S., Butler A., Lee M.J., Wilk A.J., Darby C., Zager M. (2021). Integrated Analysis of Multimodal Single-Cell Data. Cell.

[28] Hu C., Li T., Xu Y., Zhang X., Li F., Bai J., Chen J., Jiang W., Yang K., Ou Q. (2023). CellMarker 2.0: An Updated Database of Manually Curated Cell Markers in Human/Mouse and Web Tools Based on scRNA-Seq Data. Nucleic Acids Res..

[29] Franzén O., Gan L.-M., Björkegren J.L. (2019). PanglaoDB: A Web Server for Exploration of Mouse and Human Single-Cell RNA Sequencing Data. Database.

[30] Wu H., Kirita Y., Donnelly E.L., Humphreys B.D. (2019). Advantages of Single-Nucleus over Single-Cell RNA Sequencing of Adult Kidney: Rare Cell Types and Novel Cell States Revealed in Fibrosis. J. Am. Soc. Nephrol..

[31] Liao J., Yu Z., Chen Y., Bao M., Zou C., Zhang H., Liu D., Li T., Zhang Q., Li J. (2020). Single-Cell RNA Sequencing of Human Kidney. Sci. Data.

[32] Combes A.N., Phipson B., Lawlor K.T., Dorison A., Patrick R., Zappia L., Harvey R.P., Oshlack A., Little M.H. (2019). Single Cell Analysis of the Developing Mouse Kidney Provides Deeper Insight into Marker Gene Expression and Ligand-Receptor Crosstalk. Development.

[33] Newman A.M., Steen C.B., Liu C.L., Gentles A.J., Chaudhuri A.A., Scherer F., Khodadoust M.S., Esfahani M.S., Luca B.A., Steiner D. (2019). Determining Cell Type Abundance and Expression from Bulk Tissues with Digital Cytometry. Nat. Biotechnol..

[34] Jedlicka J., Soleiman A., Draganovici D., Mandelbaum J., Ziegler U., Regele H., Wuthrich R.P., Gross O., Anders H.J., Segerer S. (2010). Interstitial Inflammation in Alport Syndrome. Hum. Pathol..

[35] Daga S., Donati F., Capitani K., Croci S., Tita R., Giliberti A., Valentino F., Benetti E., Fallerini C., Niccheri F. (2020). New Frontiers to Cure Alport Syndrome: COL4A3 and COL4A5 Gene Editing in Podocyte-Lineage Cells. Eur. J. Hum. Genet..

[36] Ying Q., Wu G. (2017). Molecular Mechanisms Involved in Podocyte EMT and Concomitant Diabetic Kidney Diseases: An Update. Ren. Fail..

[37] Hu S., Hang X., Wei Y., Wang H., Zhang L., Zhao L. (2024). Crosstalk among Podocytes, Glomerular Endothelial Cells and Mesangial Cells in Diabetic Kidney Disease: An Updated Review. Cell Commun. Signal.

[38] Li Y., Yang J., Dai C., Wu C., Liu Y. (2003). Role for Integrin-Linked Kinase in Mediating Tubular Epithelial to Mesenchymal Transition and Renal Interstitial Fibrogenesis. J. Clin. Investig..

[39] Wu X., Gao Y., Xu L., Dang W., Yan H., Zou D., Zhu Z., Luo L., Tian N., Wang X. (2017). Exosomes from High Glucose-Treated Glomerular Endothelial Cells Trigger the Epithelial-Mesenchymal Transition and Dysfunction of Podocytes. Sci. Rep..

[40] Maile L.A., Gollahon K., Wai C., Dunbar P., Busby W., Clemmons D. (2014). Blocking αVβ3 Integrin Ligand Occupancy Inhibits the Progression of Albuminuria in Diabetic Rats. J. Diabetes Res..

[41] Maile L.A., Busby W.H., Gollahon K.A., Flowers W., Garbacik N., Garbacik S., Stewart K., Nichols T., Bellinger D., Patel A. (2014). Blocking Ligand Occupancy of the αVβ3 Integrin Inhibits the Development of Nephropathy in Diabetic Pigs. Endocrinology.

[42] Rubel D., Frese J., Martin M., Leibnitz A., Girgert R., Miosge N., Eckes B., Müller G.-A., Gross O. (2014). Collagen Receptors Integrin Alpha2beta1 and Discoidin Domain Receptor 1 Regulate Maturation of the Glomerular Basement Membrane and Loss of Integrin Alpha2beta1 Delays Kidney Fibrosis in COL4A3 Knockout Mice. Matrix Biol..

[43] Liu C., Zhang Y., Gao X., Wang G. (2023). Identification of Cell Subpopulations Associated with Disease Phenotypes from scRNA-Seq Data Using PACSI. BMC Biol..

[44] Fustero-Torre C., Jiménez-Santos M.J., García-Martín S., Carretero-Puche C., García-Jimeno L., Ivanchuk V., Di Domenico T., Gómez-López G., Al-Shahrour F. (2021). Beyondcell: Targeting Cancer Therapeutic Heterogeneity in Single-Cell RNA-Seq Data. Genome Med..

[45] Haller H., Bertram A., Nadrowitz F., Menne J. (2016). Monocyte Chemoattractant Protein-1 and the Kidney. Curr. Opin. Nephrol. Hypertens..

[46] Chen Z., Zhang T., Mao K., Shao X., Xu Y., Zhu M., Zhou H., Wang Q., Li Z., Xie Y. (2021). A Single-Cell Survey of the Human Glomerulonephritis. J. Cell Mol. Med..

[47] Gerhardt L.M.S., Liu J., Koppitch K., Cippà P.E., McMahon A.P. (2021). Single-Nuclear Transcriptomics Reveals Diversity of Proximal Tubule Cell States in a Dynamic Response to Acute Kidney Injury. Proc. Natl. Acad. Sci. USA.

[48] Tajti F., Kuppe C., Antoranz A., Ibrahim M.M., Kim H., Ceccarelli F., Holland C.H., Olauson H., Floege J., Alexopoulos L.G. (2020). A Functional Landscape of CKD Entities from Public Transcriptomic Data. Kidney Int. Rep..

[49] Gainer J.V., Lipkowitz M.S., Yu C., Waterman M.R., Dawson E.P., Capdevila J.H., Brown N.J., Aask Study Group (2008). Association of a CYP4A11 Variant and Blood Pressure in Black Men. J. Am. Soc. Nephrol..

[50] Ho Y., Hu P., Peel M.T., Chen S., Camara P.G., Epstein D.J., Wu H., Liebhaber S.A. (2020). Single-Cell Transcriptomic Analysis of Adult Mouse Pituitary Reveals Sexual Dimorphism and Physiologic Demand-Induced Cellular Plasticity. Protein Cell.

